# Data from emergency medicine palliative care access (EMPallA): a randomized controlled trial comparing the effectiveness of specialty outpatient versus telephonic palliative care of older adults with advanced illness presenting to the emergency department

**DOI:** 10.1186/s12873-021-00478-4

**Published:** 2021-07-12

**Authors:** Abigail M. Schmucker, Mara Flannery, Jeanne Cho, Keith S. Goldfeld, Corita Grudzen, The EMPallA Investigators, Caroline Blaum, Jason Bischof, Kei Ouchi, Marie-Carmelle Elie, Robert Swor, Karen Jubanyik, Jeffrey T. Berger, Bharath Chakravarthy, Richelle J. Cooper, Christopher J. Coyne, Chinwe H. Ogedegbe, Isabel Castro, Holden Caplan, Simar Randhawa, Jordan Carpenter, Nikita Umale, Rebecca Murray, Matthew Shaw, Nora Daut, Jennifer Bonito, Nancy Hernandez, Julia Vargas, Alexandrea Cronin, Diana McCarthy

**Affiliations:** 1 Sidney Kimmel Medical College at Thomas Jefferson University, Philadelphia, PA USA; 2 Ronald O. Perelman Department of Emergency Medicine, NYU School of Medicine, New York University Langone Health, 227 E 30th Street, First Floor, New York, NY 10016 USA; 3 Department of Population Health, New York University School of Medicine, 227 E 30th Street, First Floor, New York, NY 10016 USA

**Keywords:** Palliative care, Randomized controlled trial, Geriatrics, Advanced cancer, End-stage organ failure, Functional decline, Patient-reported outcomes, Quality of life

## Abstract

**Background:**

The Emergency Medicine Palliative Care Access (EMPallA) trial is a large, multicenter, parallel, two-arm randomized controlled trial in emergency department (ED) patients comparing two models of palliative care: nurse-led telephonic case management and specialty, outpatient palliative care. This report aims to: 1) report baseline demographic and quality of life (QOL) data for the EMPallA cohort, 2) identify the association between illness type and baseline QOL while controlling for other factors, and 3) explore baseline relationships between illness type, symptom burden, and loneliness.

**Methods:**

Patients aged 50+ years with advanced cancer (metastatic solid tumor) or end-stage organ failure (New York Heart Association Class III or IV heart failure, end stage renal disease with glomerular filtration rate < 15 mL/min/m^2^, or Global Initiative for Chronic Obstructive Lung Disease Stage III, IV, or oxygen-dependent chronic obstructive pulmonary disease defined as FEV_1_ < 50%) are eligible for enrollment. Baseline data includes self-reported demographics, QOL measured by the Functional Assessment of Cancer Therapy-General (FACT-G), loneliness measured by the Three-Item UCLA Loneliness Scale, and symptom burden measured by the Edmonton Revised Symptom Assessment Scale. Descriptive statistics were used to analyze demographic variables, a linear regression model measured the importance of illness type in predicting QOL, and chi-square tests of independence were used to quantify relationships between illness type, symptom burden, and loneliness.

**Results:**

Between April 2018 and April 3, 2020, 500 patients were enrolled. On average, end-stage organ failure patients had lower QOL as measured by the FACT-G scale than cancer patients with an estimated difference of 9.6 points (95% CI: 5.9, 13.3), and patients with multiple conditions had a further reduction of 7.4 points (95% CI: 2.4, 12.5), when adjusting for age, education level, race, sex, immigrant status, presence of a caregiver, and hospital setting. Symptom burden and loneliness were greater in end-stage organ failure than in cancer.

**Conclusions:**

The EMPallA trial is enrolling a diverse sample of ED patients. Differences by illness type in QOL, symptom burden, and loneliness demonstrate how distinct disease trajectories manifest in the ED.

**Trial registration:**

Clinicaltrials.gov identifier: NCT03325985. Registered October 30, 2017.

## Background

Emergency departments are frequently visited by severely ill patients approaching the end of life; in a study of older adults who died between 1992 and 2006, 75% visited the emergency department during the last six months of life [1]. Although the focus of emergency medicine has traditionally been the diagnosis and treatment of acute illnesses and injuries, emergency clinicians have identified a growing need for palliative care interventions in the emergency setting to address the symptoms and stresses of advanced chronic illnesses [2, 3].

Palliative care is defined by the World Health Organization as “an approach that improves the quality of life (QOL) of patients and their families facing the problems associated with life-threatening illness, through the prevention and relief of suffering by means of early identification and impeccable assessment and treatment of pain and other problems, physical, psychosocial, and spiritual.” [4] Palliative care has been shown to improve patients’ symptoms and QOL across a broad range of serious, life-limiting illnesses. Patients receiving palliative care are often able to remain cared for and supported at home, resulting in greater patient and family satisfaction and less prolonged grief and post-traumatic stress disorder among bereaved family members [5–10]. Palliative care also decreases healthcare-related costs by reducing unnecessary hospitalizations, interventions, and avoidable ED and intensive care [11–15].

In cancer patients, randomized controlled trials of palliative care interventions have shown better QOL and mood, as well as improved symptom management and patient satisfaction, with palliative care in addition to standard care [12, 16]. Palliative care has its roots in the care of cancer patients at the end of life, but increasing studies support its potential to benefit patients with end-stage organ failure, such as chronic obstructive pulmonary disease (COPD), congestive heart failure (CHF) and end-stage renal disease (ESRD), which can also cause physical, psychosocial, and spiritual distress [17, 18].

While both cancer and end-stage organ failure patients stand to benefit from palliative care, the illness trajectories associated with advanced cancer differ from those of organ failure [19]. Cancer patients commonly have a short period of evident decline before death; however, patients with end-stage organ failure have a pattern of long-term decline with episodes of worsening and remission [18]. Efforts to integrate palliative care screening and assessment into the emergency department have yielded positive outcomes, yet further research is needed to identify specific effective interventions [20]. Understanding aspects of patients’ experiences of advanced chronic diseases, such as quality of life and symptom burden, at the time of their presentation to the emergency department could assist emergency providers as they develop patient-centered treatment plans. We are not aware of any literature describing how the distinct trajectories of advanced cancer and end-stage organ failure patients manifest in the ED.

This paper reports on a large, multicenter, parallel, two-arm randomized controlled trial in ED patients comparing two established models of palliative care: nurse-led telephonic case management and specialty, outpatient palliative care. The objectives of this paper are to: 1) report preliminary baseline demographic and QOL data for the EMPallA trial cohort, 2) identify the association between illness type and baseline QOL while controlling for other baseline factors, and 3) explore the baseline relationships between illness type, symptom burden, and loneliness.

## Methods

### Study design

The Emergency Medicine Palliative Care Access (EMPallA) trial was approved by the Institutional Review Boards (IRBs) across all study sites. This study is funded through the Patient-Centered Outcomes Research Institute (PCORI). We registered this randomized controlled trial (RCT) in the international trial register (ClinicalTrials.gov: Identifier NCT03325985). This study adheres to Consolidated Standards of Reporting Trials (CONSORT) guidelines. See Supplement 1 for CONSORT checklist. For further details please refer to our protocol paper [21].

### Study setting and population

This RCT began recruitment in April 2018 and is currently enrolling at 18 emergency department (ED) sites across the United States (US), with locations representing the geographic diversity of the country. Patients considered for enrollment in this study comprise adults aged 50 years or older, who have advanced cancer (metastatic solid tumor) or end-stage organ failure (New York Heart Association class III or IV heart failure, end stage renal disease with glomerular filtration rate < 15 mL/min/m^2^ or on dialysis, or global initiative for chronic obstructive lung disease stage III, IV or oxygen-dependent), reside within the geographical area, have a working telephone, and have health insurance. Exclusion criteria include not speaking English or Spanish, having dementia documented in the electronic health record (EHR) problem list, having received hospice services or two or more palliative care visits in the last six months, residing in a long-term care facility, or being admitted to the hospital for more than 48 h post ED encounter.

### Study protocol

Enrollment of patients is ongoing as our target sample size is 1350 patients, but for this cohort occurred between April 2018 and April 3, 2020. Screening and enrollment take place seven days per week, 24 h per day. Research assistants (RAs) check the ED and observation unit electronic track boards to identify patients with qualifying medical conditions, and then review the patients’ EHR to confirm inclusion criteria are met. RAs then approach patients and conduct face-to-face interviews to confirm all eligibility criteria are met. RAs complete written informed consent and Health Insurance Portability and Accountability Act authorization and conduct a survey to gather baseline data. For Spanish-speakers, a language-appropriate consent form is used and either bilingual, certified study staff or non-investigator, hospital-employed, trained interpreters assist in acquisition of informed consent. Once enrolled, the coordinating site performs two-arm randomization (outpatient specialty palliative care vs telephonic nurse-delivered palliative care) stratified by site and illness type (cancer vs. end-stage organ failure).

The central team ensures uniform application of the methods across sites via an initial site visit, detailed Standard Operating Procedures (SOPs), standardized training of all new staff, as well as consistent re-training, and practice of the study pitch via tele-conference with central team members. Data collection, management and randomization occur using in a central REDCap database hosted at NYU School of Medicine to ensure consistency [22, 23]. Additional details are highlighted in the study protocol paper [21].

### Measures

Research assistants collect primary and secondary outcome data as well as demographic variables via face-to-face bedside interview or EHR at baseline. Demographic data include sex, race, ethnicity, income, education, religion, marital status, insurance, birth country, residency type, and language.

The primary study outcome is change in patient QOL from enrollment to six months, as measured by the Functional Assessment of Cancer Therapy-General (FACT-G, Version 4). QOL is a well-established outcome measure in palliative care research, [24, 25] and the FACT-G has been validated and used extensively to assess chronic disease therapy in many serious illnesses [24]. It has 28 items in a five-point Likert scoring scale from “0” (Not at all) to “4” (Very much) which assess QOL across four domains: physical, social/family, emotional, and functional. We followed the standard FACT-G Version 4 scoring algorithm to obtain both subscale and total FACT-G scores. We broke down FACT-G scores by domain to obtain a subscale score (possible range 0–28 for physical, social/family, and functional; 0–24 for emotional), and summed the subscale scores to obtain an overall QOL score (possible range 0–108). Higher FACT-G scores in each domain as well as overall indicate better QOL.

Secondary outcomes in patients include loneliness, as measured by change in Three-Item UCLA Loneliness Scale from enrollment to 6 months [26, 27]; and symptom burden, as measured by change in Edmonton Revised Symptom Assessment Scale (ESAS-r) from enrollment to 6 months [28]. The Three-Item Loneliness Scale comprises a three-by-three Likert scale, with higher score indicating more loneliness. To maximize validity of a three-item scale, we assessed each question (lacking companionship, feeling left out, feeling isolated from others) separately and did not sum the responses for a total loneliness score.

The ESAS-r comprises nine common symptoms rated in severity on an 11-point scale plus space to rate additional symptoms; higher scores indicate greater symptom burden. Studies of the older version of the same scale (ESAS) have shown that a more general picture of symptom severity can be gained by summing individual symptom scores for a total symptom burden score, reported on a scale from 0 to 100. Total symptom burden can therefore be assessed as absent (0), mild [1–30], moderate (31–60), or severe (61–100), and clinical significance is previously defined as a total symptom score of 31 or greater [29, 30]. For the purposes of this baseline analysis, we summed items to obtain a total symptom score but did not look at individual symptom responses (e.g. pain).

### Data analysis

We described baseline socio-demographic characteristics and functional status for patients enrolled in the study through April 2020. Means and standard deviations were calculated for continuous variables, and frequencies and percentages were calculated for categorical variables.

To assess the strength of the relationship of disease condition and QOL as measured by the FACT-G, we first used data visualization. We estimated a linear regression model to examine the importance of illness group membership in predicting overall FACT-G score, adjusting for age, education level, race, sex, immigrant status, the presence of a caregiver, comorbid illness, and hospital setting. All covariates were pre-determined a priori and included in the model based on our belief that they might be potential predictors of FACT-G scores.

Chi-square tests of independence and additional plots were used to assess the strength of the relationship between illness group and the two secondary outcomes, loneliness and symptom burden.

All analyses were conducted using R, Version 3.6.3 (R Foundation for Statistical Computing).

## Results

### Participant characteristics

**Fig. 1:**
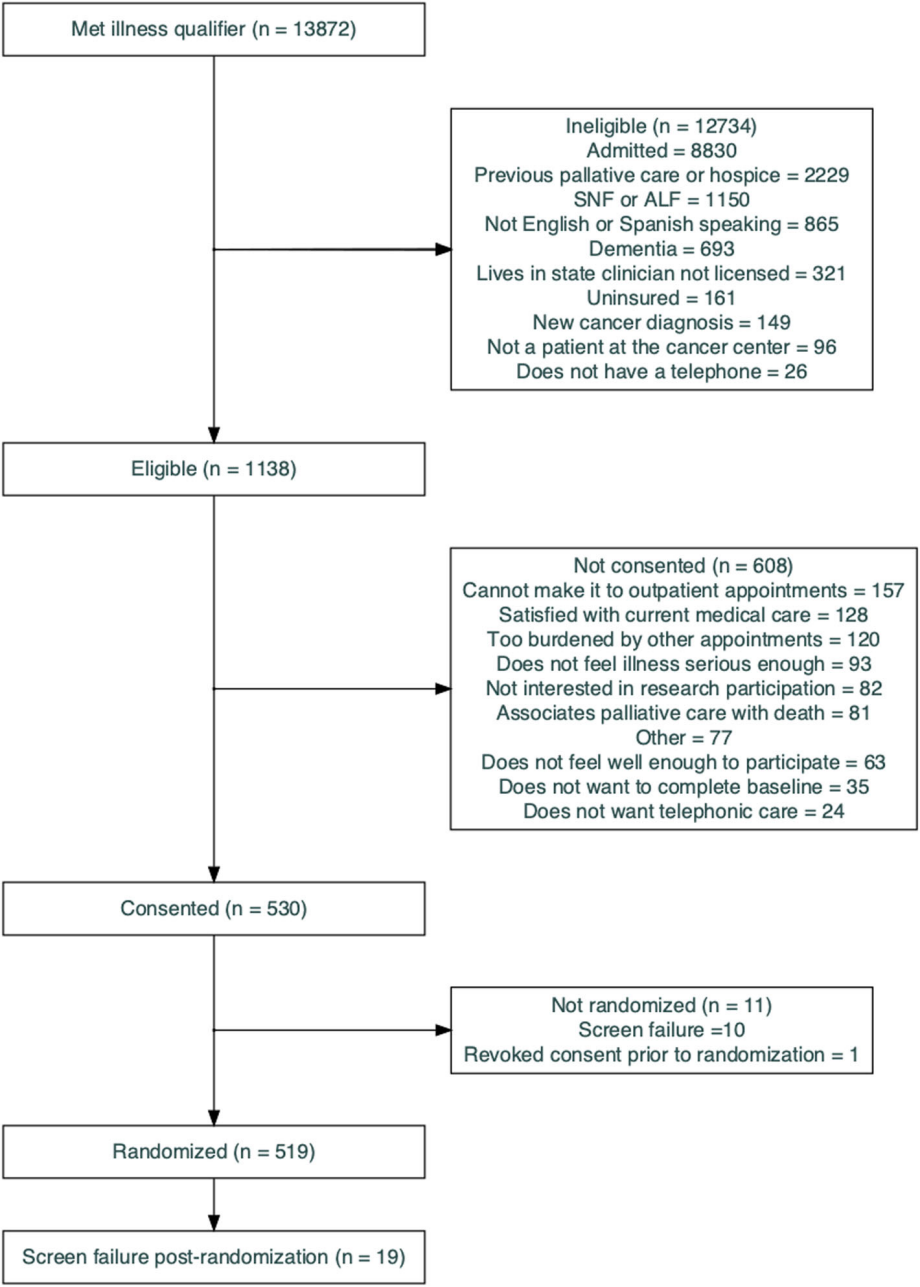
Consolidated Standards of Reporting Trials (CONSORT) Diagram

Patients recruited through April 3, 2020 were eligible for inclusion. We identified 13,872 patients with advanced illness criteria across 18 ED sites nationwide who were assessed further for eligibility. Of those patients, 1138 (8%) met all inclusion and no exclusion criteria and were eligible; 530 (47%) of those eligible were enrolled, and 519 were randomized (Fig. 1).

Ten patients were found to meet exclusion criteria after enrollment and before randomization and were never randomized. Nineteen of those randomized were determined to be screen failures post-randomization, and were excluded from the analysis. Thus, the baseline analysis and tables comprise a total of 500 study patients.

### Participant demographics

Baseline demographic characteristics are listed in Table 1. The cohort is 54% female, with a median age of 66 (IQR: 50 to 95) years, two-thirds white, 88% non-Hispanic, 89% United States-born, and 56% have at least some college education. Approximately three-quarters practice a religious faith. Thirty-eight percent have an annual household income of less than $25,000, and 32% are insured through Medicare only; 18% are dual eligible Medicare/Medicaid.

**Table 1:** Baseline Characteristics of Participants

Variable	Totals (***N*** = 500, %)
Age (50–95)	67 (10) (mean, SD)
Sex	
Female	270 (54)
Male	230 (46)
Illness*	
Cancer	206 (41)
End-stage renal disease	101 (20)
Chronic obstructive pulmonary disease	135 (27)
Congestive heart failure	125 (25)
Comorbid (two or more of above Illnesses)	
Yes	63 (13)
No	437 (87)
Race (4 Refused)	
White	294 (59)
Black	147 (29)
Other	44 (9)
Multiple races	11 (2)
Ethnicity (5 Refused)	
Hispanic	54 (11)
Non-Hispanic	441 (88)
Functional Status (2 Refused)	
Disabled	35 (7)
Requires considerable assistance	83 (17)
Requires occasional assistance	137 (27)
Cares for self, unable to do normal activity	107 (21)
Normal activity	136 (27)
Primary Language	
English	489 (98)
Spanish	11 (2)
Income (30 Refused, 53 Don’t Know)	
Less than $25 K	191 (38)
$25 K - $49,999 K	101 (20)
$50 K - $99,999 K	69 (14)
$100 K or more	56 (11)
Education Level (1 missing, 15 Refused)	
< High school degree	75 (15)
High school degree	127 (25)
Some college/AA degree	144 (29)
College degree or >	138 (28)
Marital Status (4 Refused, 1 Other)	
Married	180 (36)
Never married	111 (22)
Widow (er)	83 (17)
Separated	24 (5)
Divorced	77 (15)
Living with a partner	20 (4)
Residence type	
Apartment, elevator	125 (25)
Apartment, no elevator	79 (16)
Private home with stairs	194 (39)
Private home with no stairs	81 (16)
Other	21 (4)
Insurance Type (can have multiple)	
Employer	49 (10)
Purchased	13 (3)
Medicare	158 (32)
Medicaid	65 (13)
Veterans Affairs	3 (1)
Other	18 (4)
Dual eligible Medicare/Medicaid	89 (18)
Dual eligible (any other than Medicare/Medicaid)	105 (21)
Religion (11 Refused)	
Do not practice/believe	138 (28)
Catholic	119 (24)
Other	115 (23)
Protestant	96 (19)
Jewish	21 (4**)**
Born in US (1 Refused)	
Yes	447 (89)
No	52 (10)
Has primary family caregiver	
Yes	250 (50)
No	250 (50)
Hospital setting	
Urban	346 (69)
Suburban	154 (31)

*Patients can have multiple illnesses

Approximately one-third are married, half are private home-dwellers, and about half (51%) require assistance to care for themselves. Fifty percent have a primary family caregiver, which is defined as someone who provides regular assistance to the patient and is either a family member or a close friend who lives with the patient full-time [31].

### Data Missingness

For the demographic data, missing data was largely limited to annual household income, where 17% (*n* = 83) of the patients either did not know or declined to report it. There were some other demographic items that patients declined to report, including level of education (*n* = 15), religious status (*n* = 11), ethnicity (*n* = 5), race (*n* = 4), marital status (n = 4), functional status (*n* = 2), and birth country (n = 1).

There was virtually no missing data for the baseline survey data, where less than 1% (n = 5) of patients did not provide sufficient data to calculate an overall FACT-G score. Due to time constraints in the ED setting, two patients did not complete any questions in the UCLA 3-item loneliness scale, three additional patients declined to answer the question ‘I have a lack of companionship’, one patient declined to answer ‘I feel left out’, and one declined to answer ‘I feel isolated from others’. Also due to time constraints in the ED setting, one patient did not complete any questions in the ESAS-r.

### Baseline primary outcomes

**Fig. 2:**
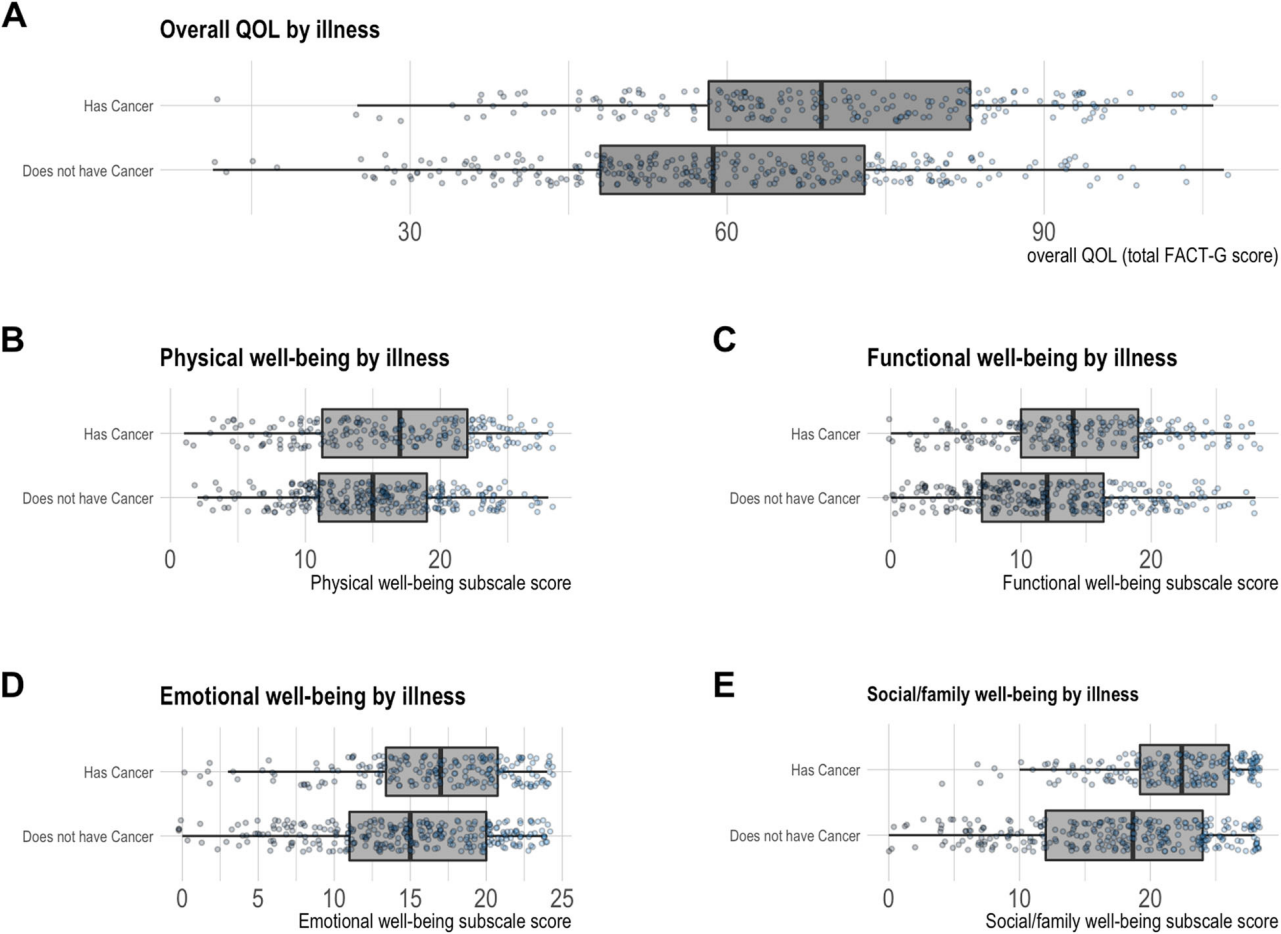
Baseline Quality of Life of Participants

Out of a possible overall FACT-G score of 108, our cohort of persons presenting to the ED with end-stage illness have an overall median FACT-G score of 63 (IQR: 11 to 107) indicating compromised QOL. The subgroup of patients with advanced cancer have less compromised QOL across all four domains (physical, emotional, social/family, and functional) as well as a less compromised total score compared to patients with end-stage organ failure (Fig. 2). Those with end-stage organ failure have a median score of 59 (11 to 107), while those with cancer have a median score of 69 (12 to 106).

The linear regression model (Table 2) based on 475 patients with complete data suggests that illness type is indeed associated with QOL after adjusting for age, education level, race, sex, immigrant status, the presence of a caregiver, and hospital setting. On average, those with end-stage organ failure have lower QOL compared to those with advanced cancer, with an estimated difference of 9.6 points (95% CI: 5.9, 13.3), and those with at least two life-limiting illnesses have a further reduction of 7.4 points (95% CI: 2.4, 12.5). This estimated difference between cancer and end-stage organ failure in overall score is consistent with the mean difference in Fig. 2. Older adults on average report better QOL than those of younger age after adjusting for all other characteristics; for each additional year of age, there is on average a 0.24 point increase (95% CI: 0.07, 0.41) in FACT-G score. The parameter estimates for the remaining characteristics were less conclusive. Overall, 8% of the variability in the outcome is accounted for by the set of covariates in our model.

**Table 2:** Linear regression to examine the importance of illness group membership in predicting FACT-G score [32].

	*Dependent variable:*
	Total FACT-G score
Age	0.24 (0.07, 0.41)
Some college or more	0.95 (−2.45, 4.35)
Has a caregiver	1.28 (−2.03, 4.58)
Suburban hospital setting	3.78 (−0.002, 7.57)
Non-white	1.84 (−1.96, 5.64)
Male	1.38 (−1.91, 4.67)
Foreign born	1.32 (−4.38, 7.01)
Not comorbid	7.42 (2.40, 12.45)
Has cancer	9.62 (5.90, 13.34)
Constant	49.71 (43.43, 55.98)
Observations	475
R^2^	0.10
Adjusted R^2^	0.08
Residual Std. Error	17.78 (df = 465)
F Statistic	5.58 (df = 9; 465)

### Baseline secondary outcomes

**Fig. 3:**
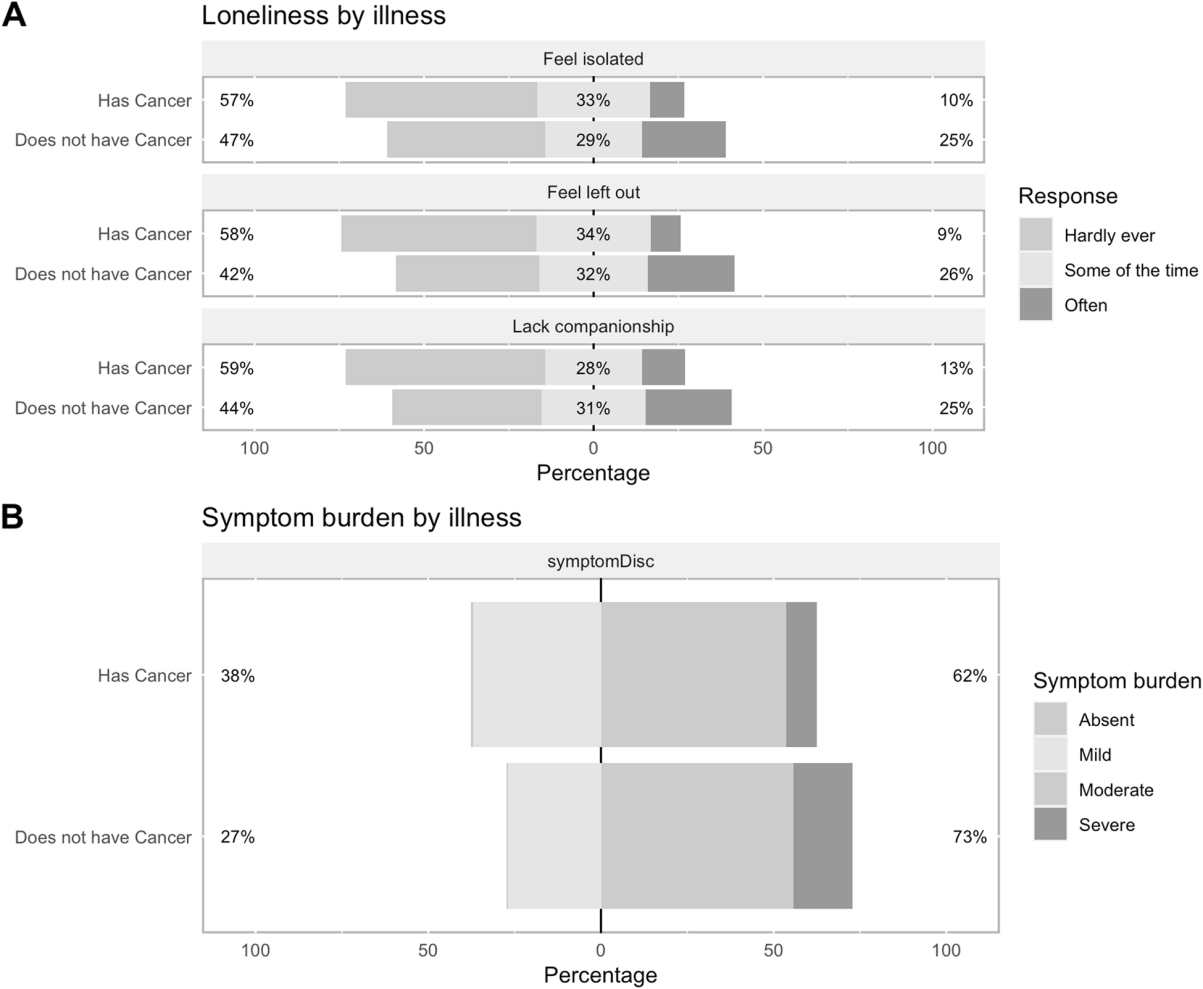
Secondary Outcomes by Illness Type

Each of the three questions on the UCLA 3-item loneliness scale was examined separately (Fig. 3 - Panel A). Ten percent of cancer patients often felt isolated, versus 25% of end-stage organ failure patients. Similar patterns were seen for the other two loneliness items (9% versus 26% often felt left out; 13% versus 25% lacked companionship). Patients with cancer appear to be more likely to have mild symptom burden (37% vs 27%) and less likely to have severe symptom burden compared with end-stage organ failure patients (9% vs 17%) (Fig. 3 - Panel B).

## Discussion

This report demonstrates that the EMPallA randomized controlled trial is enrolling a diverse sample of older adults with advanced illness who present to the ED. In the EMPallA trial cohort, 59% of patients identified as white and 11% identified as Hispanic or Latino. Female participants made of 54% of the cohort. Thirty-two percent of study participants had Medicare only, and 18% were dual-eligible for Medicare and Medicaid. Participants were distributed between the diseases of interest; cancer was the most common diagnosis at 41% and ESRD the least common at 20%. Notably, the EMPallA trial includes patients with multiple chronic conditions, whom are often excluded from randomized controlled trials [33]. Thirteen percent of study participants met eligibility criteria for two or more serious, life-limiting illnesses, and we suspect that many more have multiple conditions that are less severe. The demographics of the patients in the EMPallA study are similar to those reported in 2017 by the Centers for Disease Control and Prevention from a representative sample of ED visits in the US [34].

In this large, multi-center randomized controlled trial in ED patients, we observed important differences in baseline QOL when stratifying by disease type and controlling for demographic variables. QOL was low for all study participants, which is consistent with their advanced illness status and suggests they may particularly benefit from palliative care interventions. When comparing illness types, patients with organ failure had worse QOL on all FACT-G subscales and the overall FACT-G score as compared with cancer patients. Additionally, patients with organ failure had higher symptom burden and more loneliness than cancer patients. One reason this might be the case is that cancer patients presenting to the ED have previously been healthy and are just starting the rapid end-of-life decline in functional status, whereas organ failure patients have experienced repeated episodes of exacerbations and remissions [19]. Patients with CHF and COPD have been shown to have more hospital admissions and ED visits than cancer patients in the last months of life, which could contribute to poorer QOL [35]. Similarly, patients with ESRD tend to have lower rates of advance care planning, higher treatment intensity, and similarly high symptom burden in the last year of life compared to cancer patients [36].

Our model also indicated that meeting inclusion criteria for multiple advanced illnesses was independently associated with a poorer QOL. This is consistent with prior studies; a meta-analysis of 74 studies showed that an individual’s QOL decreases with each additional illness [37]. Additionally, we found that older age is associated with improved QOL. We are unsure about why this is, but hypothesize it might be because patients who have lived longer have likely experienced less serious illness during their lifetime.

To our knowledge, this is the first large, multicenter trial enrolling older adults with multiple different advanced illnesses from the ED. While the distinct disease trajectories of patients with cancer and end-stage organ failure have been elucidated in other settings, this report is unique in presenting a snapshot of the distinct trajectories at the time of an ED visit. The differences by illness type in QOL, symptom burden, and loneliness seem to reflect the sudden deterioration of cancer patients at the end of life versus the undulating decline of those with organ failure [19].

For older adults with serious illness presenting to the ED, we have demonstrated that QOL, symptom burden, and loneliness differ by illness type, suggesting that palliative care needs may differ by disease category as well [38]. For this reason, the subgroup analyses by illness type in the final EMPallA analysis will be important in determining how to interpret the primary outcome of the study for patients of different illness types and in designing future palliative care interventions that benefit particular disease types. Despite similar symptom burdens, illness experience, and survival rates between advanced cancer and end-stage organ failure, much of the attention directed toward improving end-of-life care has focused on cancer patients [17, 35, 36, 39]. This report highlights the need for more disease-specific research to improve patient-centered outcomes for those with end-stage organ failure presenting to the ED. Additionally, since patients with multiple chronic conditions have particularly low QOL, future research should focus on developing palliative care interventions to target these patients, as well as screening and referral protocols to be used by ED providers.

A strength of our study is that it is enrolling a large national cohort with a rich set of baseline demographic variables and patient-reported outcome measures. Its inclusive inclusion criteria facilitate recruitment of diverse patients. Additionally, by including patients with both cancer and end-stage organ failure, palliative care interventions can be assessed in unique disease processes.

Nevertheless, our study has various limitations. Only about 50% of patients who are approached are willing to enroll in the study. Patient refusal or failure to follow up could result in a non-response bias, where enrolled patients are those open to trying palliative care. Additionally, this study is limited to four specific diseases, only recruits English- and Spanish-speaking patients, and is conducted within the US, meaning it may not be generalizable to patients outside these demographics. Finally, all outcomes are self-reported rather than objectively measured. However, patient-reported outcomes are commonly used in palliative care and are validated to measure QOL and symptom burden [24, 25].

## Conclusions

The EMPallA trial is enrolling a diverse sample of ED patients. The lower QOL, increased symptom burden, and increased loneliness found in end-stage organ failure patients versus cancer patients demonstrates how distinct disease trajectories manifest in the ED.

## Data Availability

The data that support the findings of this study are available from PCORI but restrictions apply to the availability of these data, which were used under license for the current study, and so are not publicly available. Data are however available from the authors upon reasonable request and with permission of PCORI.

## Abbreviations

ED: Emergency department
QOL: Quality of life
FACT-G: Functional Assessment of Cancer Therapy-General
EMPallA: Emergency Medicine Palliative Care Access
COPD: Chronic obstructive pulmonary disease
CHF: Congestive heart failure
ESRD: End-stage renal disease
IRB: Institutional review board
PCORI: Patient-Centered Outcomes Research Institute
RCT: Randomized controlled trial
US: United States
HER: Electronic health record
RA: Research assistant
ESAR-r: Edmonton Revised Symptom Assessment Scale
